# Training the Workforce in Evidence-Based Public Health: An Evaluation of Impact Among US and International Practitioners

**DOI:** 10.5888/pcd10.130120

**Published:** 2013-09-05

**Authors:** Wesley S. Gibbert, Shannon M. Keating, Julie A. Jacobs, Elizabeth Dodson, Elizabeth Baker, Gunter Diem, Wayne Giles, Kathleen N. Gillespie, Vilius Grabauskas, Aushra Shatchkute, Ross C. Brownson

**Affiliations:** Author Affiliations: Shannon M. Keating, Health Capital Consultants, St. Louis, Missouri; Julie A. Jacobs, Elizabeth Dodson, The Prevention Research Center in St. Louis at the Brown School at Washington University in St. Louis, St. Louis, Missouri; Elizabeth Baker, The Prevention Research Center in St. Louis and Saint Louis University School of Public Health, St. Louis, Missouri; Gunter Diem, Vorarlberg Public Health Society, Vorarlberg, Austria; Wayne Giles, Centers for Disease Control and Prevention, Atlanta, Georgia; Kathleen N. Gillespie, Saint Louis University School of Public Health, St. Louis, Missouri; Vilius Grabauskas, Lithuanian University of Health Sciences, Kaunas, Lithuania; Aushra Shatchkute, formerly affiliated with the World Health Organization Countrywide Integrated Noncommunicable Disease Intervention Program, Copenhagen, Denmark; Ross C. Brownson, The Prevention Research Center in St. Louis at the Brown School at Washington University in St. Louis and Washington University School of Medicine, Washington University in St. Louis, St. Louis, Missouri. Ms. Jacobs is now a public health consultant in Lexington, Kentucky.

## Abstract

**Introduction:**

The Prevention Research Center in St. Louis developed a course on evidence-based public health in 1997 to train the public health workforce in implementation of evidence-based public health. The objective of this study was to assess use and benefits of the course and identify barriers to using evidence-based public health skills as well as ways to improve the course.

**Methods:**

We used a mixed-method design incorporating on-site pre- and post-evaluations among US and international course participants who attended from 2008 through 2011 and web-based follow-up surveys among course participants who attended from 2005 through 2011 (n = 626). Respondents included managers, specialists, and academics at state health departments, local health departments, universities, and national/regional health departments.

**Results:**

We found significant improvement from pre- to post-evaluation for 11 measures of knowledge, skill, and ability. Follow-up survey results showed at least quarterly use of course skills in most categories, majority endorsement of most course benefits, and lack of funding and coworkers who do not have evidence-based public health training as the most significant barriers to implementation of evidence-based public health. Respondents suggested ways to increase evidence-based decision making at their organization, focusing on organizational support and continued access to training.

**Conclusion:**

Although the evidence-based public health course is effective in improving self-reported measures of knowledge, skill, and ability, barriers remain to the implementation of evidence-based decision making, demonstrating the importance of continuing to offer and expand training in evidence-based public health.

## Introduction

Public health is a global, transdisciplinary field, drawing a workforce from various backgrounds (1,2). Practitioners include physicians, nurses, health educators, biostatisticians, epidemiologists, environmental health officers, policy makers, and other professionals who support the mission to protect, promote, and improve the health of the public (3,4). Workforce-related challenges in public health include a lack of formal training and frequent staff turnover (5,6). The diversity of backgrounds and education of public health workers calls for educational offerings to develop a common base of understanding of the fundamentals of public health practice (3).

Evidence-based public health (EBPH) is defined as the process of integrating evidence from scientific research with community preferences to improve population health (7). Recent efforts have established more uniform EBPH training guidelines for public health practitioners. In the United States, the Institute of Medicine released recommendations for skills, which evolved from the Core Competencies for Public Health Professionals (8). Additionally, through the Public Health Accreditation Board (PHAB), voluntary accreditation is available for tribal, local, and state health departments. The PHAB established 12 domains of achievement, including one that specifies the importance of using evidence in public health practice (9). The World Health Organization also focuses on the composition and training of the public health workforce (2,10). EBPH courses can improve the capacity of the public health workforce (11–13).

To meet the training needs of the state and local public health workforce, the Prevention Research Center in St. Louis (PRC–StL) began an EBPH course in 1997 and in 2002 expanded the course to Europe in cooperation with the Collaboration for Integrated Noncommunicable Disease Intervention Programme (11). The course covers essential EBPH concepts in 9 modules during 3 or 4 days, integrating group exercises and demonstrations. Course content is based on the key components of EBPH, which align closely with many core competencies of public health (5–10). Course content was designed to meet the training needs and required skills and competencies of public health practitioners (11,14,15). The PRC-StL’s EBPH course was one of the first to tailor and teach competency-based EBPH principles directly to public health practitioners outside of the schools of public health, bringing needed workforce development to practitioners who often do not have training in one or more core public health disciplines (eg, epidemiology, behavioral science) (2,3,11,12,14–16). Courses, including state courses, national courses, and a yearly international course, are held multiple times per year in various settings. Participants apply to a course partner or the PRC and are selected according to criteria that include room in the course, applicant job title, and previous training. The course has reached more than 1,000 participants representing all 50 US states, 38 countries, and 4 continents.

In a 2001–2004 evaluation of course participants, 90% of respondents indicated that the course helped them make more informed decisions in the workplace, communicate more effectively with coworkers, and better understand reports (12). The course was associated with creation of a common knowledge base for public health practitioners and enhanced ability to garner funding (13).

The objective of this mixed-method evaluation was to assess the EBPH course among a new set of participants by examining their use of the training, the benefits of and barriers to using the training, and how those differ by location, education, and other characteristics. We measured participants’ skills and development and identified barriers to using EBPH skills with the goals of improving the course and assessing changes in evidence-based practice since the previous evaluation (12).

## Methods

### Evaluation design, measures development, and sampling

A mixed-method study design incorporated on-site evaluations at each course from 2008 through 2011 and off-site follow-up surveys sent to all course participants from 2005 through 2011. We administered pre- and post-evaluations to 393 participants at 15 EBPH courses offered from 2008 through 2011 to assess the immediate impact of the course; these on-site evaluations were not available from 2005 through 2007. We asked participants to report their knowledge, skill, or ability level for 11 items on a 5-point Likert scale (from strongly disagree to strongly agree) immediately before and after the course.

We sent a follow-up survey (distinct from the on-site pre- and post-evaluation instrument) to all participants (n = 626) in courses from May 2005 to December 2011, including those who had completed the on-site evaluation. Names of participants in 26 EBPH courses (20 US and 6 international) were compiled into a master list of eligible participants, each of whom received an e-mail explaining the study and providing a link to the secure online survey; e-mail addresses were provided during course registration. In June 2009, 217 US course participants received the follow-up survey, followed by 87 international course participants in December 2009, and 322 additional US and international course participants in June 2012. Each survey was open for approximately 2 months, and we sent 2 or 3 reminder e-mails. US participants in 2009 received reminder telephone calls at work, if necessary; telephone numbers were provided during course registration.

The 15-question online survey was adapted from a tool developed collaboratively by course instructors for a previous evaluation (12). To assess the effectiveness of the EBPH course in increasing evidence-based practice, survey questions addressed frequency of use of EBPH course content, benefits and barriers to use, and use of evidence-based programs in respondents’ agencies. We used a 5-point Likert scale to assess benefits and barriers. We asked 2 open-ended questions. One asked, “In your opinion, what is the one thing that most needs to happen at your agency to increase the use of evidence-based decision making?” The other asked, “What one thing could be done to improve the evidence-based public health training?”

### Analyses

We used paired-samples *t* tests to analyze the on-site pre- and post-evaluations. We used descriptive statistics, χ^2^ tests, independent-samples *t* tests, and analysis of variance to analyze quantitative data from the follow-up survey. Data were stratified by course location (United States, international), agency type (state health department, city or county health department, national or regional health department, university, other), highest level of education (BA/BS, MPH/MSPH, MS/MSc/MA/other master, MD/DO/PhD/DrPH/ScD, RN/RD/NP), job type (specialist, manager, academic, other), and years of experience in public health (0–4.9 y, 5–9.9 y, 10–17.9 y, 18–37.5 y) to discern differences in the use of and benefits and barriers to use of course content. We categorized use of course content into 2 dichotomous variables (used at least monthly and used at least quarterly). We examined benefits of and barriers to use of course content as the percentage of respondents who agreed or strongly agreed (4 or 5 on the 5-point Likert scale) to the survey statements. US participants’ agencies were categorized as state or local (ie, city or county) health departments.

Participants’ responses to the 2 open-ended questions were analyzed by using focused coding qualitative analysis (17). Responses from the 2009 and 2012 surveys were analyzed separately by 2 sets of authors (S.M.K./E.D. and W.S.G./E.D., respectively). The resulting categories for each survey were then compared, and consensus was reached (by creating new categories or combining existing categories) when discrepancies were identified. Data from all surveys were then combined.

## Results

### On-site course evaluation

Pre- and post-evaluations were given to 393 course participants between 2008 and 2011 and completed by 352 participants for a response rate of 89.6%. We found significant increases from pre- to post-evaluation for all 11 measures (*P* < .001). Having a good enough understanding of economic evaluation to assist in the design and implementation of an economic evaluation was most improved (0.77 point increase on a 5-point Likert scale), whereas the ability to develop a concise statement of the issue was least improved (0.47 point increase).

### Quantitative follow-up survey

The follow-up surveys were completed by 358 participants (response rate 57.2%) (Table 1). US respondents represented 82.7% of the sample, whereas international respondents, mainly from central and eastern Europe, represented 17.3%. US and international respondents differed significantly by highest level of education attained (*P* < .001); all international respondents reported postgraduate training. Most US respondents worked for state health departments (72.4%), whereas international respondents primarily worked for national or regional health departments (36.0%). Most respondents (56.7%) worked in managerial positions. Respondents were more likely to have a graduate degree in something other than public health (61.7%) than public health (18.1%).

**Table 1:** Characteristics of Participants in Online Survey of Evidence-Based Public Health Course (N = 358), 2005–2011^a^

Characteristic	All	United States	International
**Location**			
United States	296 (82.7)	—	—
International	62 (17.3)	—	—
**Agency^b^**			
No. of respondents	289	239	50
State or local health department	178 (61.6)	173 (72.4)	5 (10.0)
City or county health department	33 (11.4)	30 (12.6)	3 (6.0)
National or regional health department	18 (6.2)	—b	18 (36.0)
University	25 (8.7)	15 (6.3)	10 (20.0)
Other	35 (12.1)	21 (8.8)	14 (28.0)
**Highest degree**			
No. of respondents	277	229	48
MD, DO, PhD, DrPH, or ScD	62 (22.4)	32 (14.0)	30 (62.5)
MPH or MSPH	50 (18.1)	44 (19.2)	6 (12.5)
MS, MSc, MA, or other master	86 (31.0)	74 (32.3)	12 (25.0)
RN, RD, or NP	23 (8.3)	23 (10.0)	0
BA or BS	56 (20.2)	56 (24.5)	0
**Job type**			
No. of respondents	289	241	48
Specialist^c^	76 (26.3)	66 (27.4)	10 (20.8)
Manager^d^	164 (56.7)	142 (58.9)	22 (45.8)
Academic	21 (7.3)	11 (4.6)	10 (20.8)
Other	28 (9.7)	22 (9.1)	6 (12.5)
**Years in public health, mean (SD)**	12.1 (8.4)	12.4 (8.6)	10.6 (7.3)

Abbreviations: MD, doctor of medicine; DO, doctor of osteopathic medicine; PhD, doctor of philosophy; DrPH, doctor of public health; ScD, doctor of science; MPH, master of public health; MSPH, master of science in public health; MS, MSC, master of science; MA, master of arts; RN, registered nurse; RD, registered dietitian; NP, nurse practitioner; BA, bachelor of arts; BS, bachelor of science. Dashes (—) indicate that category does not apply.

a All values are numbers (percentages) unless otherwise indicated. Not all respondents answered all questions.

b US survey participants were given the following categories: state health department, city or county health department, university, and other. International participants were given the following: national or regional health department; university; state or local health department, and other.

c Specialist includes health educator, epidemiologist, statistician, program planner, program evaluator, community health nurse, social worker, dietitian, and nutritionist.

d Manager includes program manager, administrator, or coordinator, division or bureau head, division deputy director, and department head.

Respondents reported high levels of use of EBPH course materials. Most respondents reported at least quarterly use of EBPH course materials in 5 of 6 categories. A quarter of respondents reported at least monthly use in 3 of 6 categories (Table 2). Use differed significantly by location and agency type in some categories but not by job type. US state health department workers reported more use than local health department workers in 5 of 6 categories both monthly and quarterly. Use also differed by education level.

**Table 2:** Percentage of Respondents Indicating Agreement With Use of, Benefits of, and Barriers to Using Evidence-Based Public Health (EBPH) Course Materials, by Location (All Participants) and Agency (US Participants Only), 2005–2011

Survey Item	All Participants, % (n^a^)				US Participants Only, % (n^a^)		
	Total (n = 358)	United States (n = 296)	International (n = 62)	***P* b**	State Health Department (n = 173)	Local Health Department (n = 30)	***P* b**
**On average, every month since the EBPH course I have**							
Searched the scientific literature for information on programs.	43 (308)	41 (256)	54 (52)	.08	39 (173)	20 (30)	.04
Used the EBPH materials/skills in modifying an existing program.	25 (310)	26 (257)	25 (53)	.86	23 (173)	17 (30)	.43
Used the EBPH materials/skills in evaluating a program.	27 (306)	26 (254)	33 (52)	.29	23 (171)	27 (30)	.64
Used the EBPH materials/skills in planning a new program.	22 (309)	21 (256)	26 (53)	.40	19 (172)	17 (30)	.80
Referred to the EBPH readings that were provided.	15 (308)	12 (256)	31 (52)	<.001	10 (172)	3 (30)	.22
Used the EBPH materials/skills for grant applications.	9 (307)	9 (254)	9 (53)	>.99	8 (171)	3 (30)	.35
**The EBPH course content helped me**							
See applications for this knowledge in my work.	81 (301)	82 (249)	79 (52)	.60	81 (173)	73 (30)	.34
Become a better leader who promotes evidence-based decision making.	79 (299)	80 (248)	73 (51)	.25	80 (173)	73 (30)	.38
Acquire knowledge about a new subject.	79 (303)	78 (250)	85 (53)	.29	80 (173)	63 (30)	.04
Make scientifically informed decisions at work.	72 (302)	74 (249)	64 (53)	.15	72 (173)	67 (30)	.53
Implement evidence-based practices in CDC cooperative agreement or other funded programs.	59 (266)	60 (248)	50 (18)	.40	62 (173)	43 (30)	.06
Communicate better with coworkers.	61 (297)	59 (245)	73 (52)	.05	59 (172)	43 (30)	.10
Teach others how to use/apply the information in the EBPH course.	58 (299)	58 (248)	61 (51)	.68	56 (170)	57 (30)	.98
Read reports and articles.	57 (299)	57 (247)	58 (52)	.94	55 (173)	43 (30)	.24
Develop a rationale for a policy change.	53 (298)	52 (246)	58 (52)	.49	49 (172)	53 (30)	.69
Adapt an intervention to a community's needs while keeping it evidence based.	53 (297)	51 (247)	60 (50)	.27	49 (172)	50 (30)	.91
Identify and compare the costs and benefits of a program or policy.	49 (298)	49 (247)	51 (51)	.80	49 (173)	45 (29)	.71
Prepare a policy briefing for administrators or state or local legislative officials.	31 (298)	29 (247)	41 (51)	.08	26 (172)	33 (30)	.42
Obtain funding for programs at work.	30 (300)	28 (248)	38 (52)	.14	21 (173)	50 (30)	.001
**I have not used the EBPH course content as much as I would like because**							
There is not enough funding for continued training in EBPH.	35 (293)	30 (243)	56 (50)	.001	29 (170)	33 (30)	.67
The people I work with do not have EBPH training.	32 (294)	29 (244)	44 (50)	.04	28 (171)	23 (30)	.59
I do not have enough time to implement EBPH approaches.	24 (292)	21 (242)	38 (50)	.01	20 (169)	23 (30)	.69
Within my agency there are no incentives to use EBPH.	20 (294)	19 (244)	22 (50)	.66	19 (171)	17 (30)	.73
There was too much information and not enough time to process it.	15 (295)	14 (244)	20 (51)	.30	15 (171)	20 (30)	.45
My organization does not have a culture that supports the use of EBPH approaches.	14 (293)	13 (243)	18 (50)	.32	12 (170)	13 (30)	.88
The information was too complex.	4 (293)	5 (243)	2 (50)	.41	4 (171)	7 (30)	.53
The information lacked relevance.	3 (294)	3 (244)	4 (50)	.67	4 (171)	0 (30)	.26

Abbreviation: CDC, Centers for Disease Control and Prevention.

a Not all survey respondents answered all questions. The n’s in parentheses indicate the number of respondents who answered the question. Percentages were calculated by using the number who answered the question.

b Determined by Pearson χ^2^ test.

Most respondents indicated agreement with 10 of 13 statements on benefits of EBPH course content (Table 2). Respondents with a nursing degree reported the highest level of agreement, compared with other groups of respondents, in 12 of the 13 benefit categories; their level of agreement differed significantly from respondents with other degrees in in 3 categories: read reports and articles, obtain funding for programs at work, and develop a rationale for policy change. Agreement on benefits differed significantly in several categories by US agency type (Table 2) and job type (Table 3).

**Table 3:** Percentage of Respondents Indicating Agreement With Use of, Benefits of, and Barriers to Using Evidenced-Based Public Health (EBPH) Course Materials, by Job Type, 2005–2011

Survey Item	Specialist, % (n^a^) (N = 76)	Manager, % (n^a^) (N= 164)	Academic, % (n^a^) (N = 21)	***P* Value^b^**
**On average, every month since the EBPH course I have**				
Searched the scientific literature for information on programs.	15 (75)	15 (162)	25 (20)	.51
Used the EBPH materials/skills in planning a new program.	18 (76)	28 (163)	15 (20)	.19
Used the EBPH materials/skills in modifying an existing program.	20 (76)	31 (163)	20 (20)	.13
Used the EBPH materials/skills in evaluating a program.	9 (75)	11 (161)	5 (20)	.66
Referred to the EBPH readings that were provided.	49 (76)	41 (161)	55 (20)	.33
Used the EBPH materials/skills for grant applications.	23 (75)	30 (161)	35 (20)	.38
**The EBPH course content helped me**				
Acquire knowledge about a new subject.	75 (76)	83 (163)	70 (20)	.21
See applications for this knowledge in my work.	84 (76)	83 (163)	85 (20)	.98
Become a better leader who promotes evidence-based decision making.	76 (76)	74 (163)	70 (20)	.82
Make scientifically informed decisions at work.	67 (75)	59 (160)	47 (19)	.25
Read reports and articles.	60 (75)	58 (163)	47 (19)	.60
Communicate better with coworkers.	22 (76)	33 (163)	53 (19)	.03
Identify and compare the costs and benefits of a program or policy.	43 (74)	58 (163)	63 (19)	.08
Implement evidence-based practices in CDC cooperative agreement or other funded programs.	41 (76)	55 (163)	47 (19)	.14
Teach others how to use/apply the information in the EBPH course.	24 (76)	36 (162)	26 (19)	.15
Develop a rationale for a policy change.	57 (76)	52 (162)	67 (18)	.48
Adapt an intervention to a community's needs while keeping it evidence based.	58 (76)	58 (163)	79 (19)	.19
Prepare a policy briefing for administrators or state or local legislative officials.	54 (72)	65 (148)	69 (13)	.26
Obtain funding for programs at work.	71 (76)	86 (163)	68 (19)	.01
**I have not used the EBPH course content as much as I would like because**				
Within my agency there are no incentives to use EBPH.	24 (76)	25 (159)	16 (19)	.66
There is not enough funding for continued training in EBPH.	3 (76)	6 (160)	0 (18)	.37
I do not have enough time to implement EBPH approaches.	3 (76)	3 (160)	5 (19)	.78
The people I work with do not have EBPH training.	12 (76)	17 (161)	21 (19)	.49
My organization does not have a culture that supports the use of EBPH approaches.	16 (75)	13 (160)	16 (19)	.82
There was too much information and not enough time to process it.	16 (76)	22 (160)	32 (19)	.27
The information was too complex.	34 (76)	34 (159)	53 (19)	.26
The information lacked relevance.	36 (76)	30 (160)	32 (19)	.70

Abbreviation: CDC, Centers for Disease Control and Prevention.

a Not all survey respondents answered all questions. The n’s in parentheses indicate the number of respondents who answered the question. Percentages were calculated by using the number who answered the question.

b Determined by Pearson χ^2^ test.

Generally, respondents showed a higher level of agreement with statements on barriers related to implementing evidence-based decision making than with statements on barriers related to the course itself. The most frequently cited barriers were lack of funding for continued EBPH training and working with people who do not have EBPH training. International respondents were more likely than US respondents to agree with statements on barriers; we noted 3 significant differences (Table 2). Among all respondents, those who had worked in public health either a short time (0–4.9 y) or a long time (18–37.5 y) more often agreed with the statements on barriers than those in public health for an intermediate time (5–17.9 y). Those in public health a long time (18–37.5 y) were significantly more likely than those in public health a shorter time (0–17.9 y) to report that the course presented too much information (*P* = .047) and that the information was too complex (*P* = .02). Respondents with a doctoral degree were significantly more likely than those with a nursing degree to report a lack of funding for continued EBPH training (*P* = .001); and respondents with a public health master’s degree were significantly more likely than those with a bachelor’s degree to report working with people who do not have EBPH training (*P* = .02).

Provided with a detailed definition of an evidence-based program, 248 respondents reported that an average of 62% of the programs at their agency was evidence-based (95% confidence interval [CI], 59.1%–65.6%). US respondents (n = 207) reported that 64% (95% CI, 60.2%–67.1%) of the programs in their agency were evidence-based, whereas 41 international respondents reported 56% (95% CI, 47.2%–64.5%). US state health department workers (n = 145) reported that 62% (95% CI, 57.4%–66.0%) of programs in their agency were evidence-based, whereas 27 US local health department workers reported 72% (95% CI, 65.7%–79.1%).

### Qualitative follow-up survey

The most frequently mentioned theme in response to the question on how to increase evidence-based decision making was lack of training for public health professionals. Many respondents indicated that training of all staff within an organization was particularly important. Respondents also mentioned easy, quick access to journals, articles, and more information on evidence-based practices. One respondent requested “more information about specific programs that work. The community guide is very vague. Specific programs . . . are more realistic to implement.” Respondents also stressed the importance of better means of sharing information within and across organizations. As one respondent noted, “I think we need to have better communication across units so that more people understand what resources are available to help them make evidence-based decisions.”

The next set of themes reflected barriers to implementation of evidence-based decision making. The second most frequent theme was time for implementation and practice of EBPH as well as general and program planning. One respondent noted, “For me it is the lack of time to practice these concepts. . . . Implementation is not possible when so many issues are coming at you.” The third most frequent theme was the lack of funding for evidence-based programs, staff, and evaluation. Respondents endorsed funders that require evidence-based practices.

The fourth most frequent theme was leadership. One respondent said, “Leaders at the very top need to indicate this approach is mandatory. . . . It needs to be made one of our values.” Other respondents agreed, indicating that leaders, administrators, and managers need to understand and encourage the use of EPBH within an organization. The leadership theme dovetailed with the expressed need to make EBPH part of the culture and values of an organization. The importance of increasing buy-in from coworkers was also frequently mentioned.

The main themes in response to the question on how to improve EBPH training were course follow-up, course length, and coursework level. The strongest and most frequently mentioned theme concerned the examples and activities integrated into most modules. Respondents indicated that these could be made more effective by using “one consistent area . . . throughout the modules,” choosing “one topic . . . for all examples and group work,” and using “examples that are specific to the location of the participants.” The second most frequent theme was providing postcourse follow-up. Respondents specified that follow-up sessions “that might keep it fresh in our memories” would be useful. Respondents endorsed the benefits of web-based sessions (eg, webinars, web-based mini-trainings, or online sessions). Course time was the third most frequent theme, with respondents indicating that the course was too short, with too much information “to digest in the allotted timeframe,” and suggesting “precourse reading and work to help students prepare for class.” The fourth most frequent theme was coursework level: one respondent said the coursework was “too elementary for my knowledge,” several requested more advanced coursework, and others found parts of the course too challenging.

## Discussion

Our practitioner-focused EBPH course seeks to address the need for training and education throughout the public health workforce to fill the gaps in the background of public health practitioners (5,18). This study demonstrates the effectiveness of the course, with the quantitative data showing that knowledge in all areas increased. However, the qualitative data suggest ways to improve the course, emphasizing the importance of tailoring the course to each participant group and providing reminders and access to the course information after the course.

Direct comparison between this current evaluation and the 2008 evaluation provides a unique view of the evolution of the course and EBPH from 2005 to 2011 (12). Monthly use of course skills and materials changed in different ways. Searching the scientific literature was reported most often in both evaluations but at a slightly lower level in this evaluation (55% in 2008 and 43% in this evaluation). Using EBPH materials in modifying an existing program, evaluating a program, or planning a program were all reported at slightly higher levels in this evaluation (3–7 percentage points higher). Fewer respondents reported referring to the EBPH readings in this evaluation (15% from 31%), likely reflecting presentation of the readings on compact disk rather than on paper (12). In both evaluations, barriers external to the course were reported more often than barriers directly related to course content. Interestingly, the percentage of respondents who identified job-related time constraints as a significant barrier decreased from 71% in 2005 to 24% in the current evaluation (12). Generally the course appears to have remained as effective or become slightly more effective since the 2005 evaluation.

Although support for EBPH is increasing, barriers to implementation remain (5). Organizational barriers were cited more often than personal barriers; the most frequently identified barriers were lack of funding for continued training, coworkers who do not have EBPH training, lack of time, and lack of incentives (19). The repetition of these themes in our qualitative data and the literature emphasizes their importance (6,13,19). Several of these barriers have been connected to lack of support in an organizational culture for implementing EBPH (13,19).

Respondents suggested several solutions to these barriers. Both our quantitative and qualitative findings showed the importance of leadership in fostering evidence-based decision making, consistent with previous literature (13,20,21). Leadership support and understanding are important to the implementation of not only EBPH but also evidence-based programs (22). Many leadership-training programs exist to develop a culture of evidence-based decision making (23,24).

Even in the presence of strong leadership, a critical mass and social network in support of evidence-based decision making are needed (25). The perception of institutional priority for evidence-based practices has been correlated with use of research to inform program adoption and implementation (26). Support for or requirement of EBPH practices by funders can also increase organizational support (27). The recent move toward accreditation for health departments imparts similar support (27,28).

Respondents emphasized both the need for additional types of training and training for more public health practitioners. Although evidence-based practice is moving toward a transdisciplinary model, it remains important for those offering EBPH training to recognize the breadth of experience in the public health workforce and tailor their trainings to specific groups, as reported in our qualitative data (3,5,29). Respondents requested both more basic and more rigorous training broadly and in specific areas such as economic evaluation. Despite EBPH training resources both in the United States and abroad, the second-most frequently reported barrier to implementing EBPH course content was coworkers who had no EBPH training, suggesting the importance of expanding existing EBPH trainings, creating new EBPH trainings, and further integrating EBPH coursework into academic public health programs at multiple levels (27,30). All 50 US states could use funding available from the Coordinated Chronic Disease Prevention and Health Program for chronic disease prevention training to expand EBPH training (31). Recent recommendations based on growing evidence point to web-based training as important for future efforts (32). Administrative evidence-based practices like expansion and tailoring of EBPH training and organizational support and policies for EBPH are needed to foster EBPH (33).

Strengths of this evaluation include building on previous evaluations to discern trends in EBPH use and continuing benefits and barriers of the EBPH principles in this course as well as the use of mixed methods to triangulate results. Limitations include a relatively low response rate (possibly introducing selection bias), possible inaccuracy in self-reported data, and the inability to draw a causal relationship between course attendance and behavior change due to lack of a control group. Language may also have been a barrier in survey response for international participants, and the smaller international sample may decrease generalizability of the results for international practitioners.

This evaluation gathers new information from public health practitioners nationally and internationally. These data show that the PRC-StL’s EBPH course appears to be effective in increasing skills in and use of EBPH among course participants. The survey data build on past evaluations to gain additional insight into the remaining barriers to implementation of EBPH principles, and the qualitative data provide new strategies for overcoming those barriers. Leadership support within organizations and funder support from without are needed both to encourage the use of EBPH principles and to build organizational cultures that value EBPH. A need remains for additional and more diverse training in EBPH at national and international levels, tailoring EBPH learning to practitioners’ needs, and working toward a multi-disciplinary understanding of evidence-based practice. Courses with varying levels of rigor, specific areas of focus, and novel delivery methods, such as distance learning are warranted. There is also a need among practitioners for increased access to the scientific literature and other resources integral to evidence-based practice. This evaluation allows for the PRC-StL and others to better understand the barriers remaining to successful implementation of evidence-based practices and to take steps to overcome them.
